# The effect of simvastatin on patients with traumatic brain injury

**Published:** 2012-11

**Authors:** Saleh Rasras, Farhad Rahbariyan, Mojtaba Khezri

**Affiliations:** ^*a*^Department of Neurosurgery, Bojnord Hospital, Bojnord, Iran.

## Abstract

**Background:**

Traumatic brain injury is one of the most common causes harmful to the health of society. Several studies have been conducted on the treatment of traumatic brain injury. The protective effects of statins on neurons have been demonstrated in numerous studies. The objective of the present study is to examine the effect of simvastatin on the short-term and long-term results of consciousness in patients with brain trauma.

**Methods:**

66 patients with traumatic brain injury with GCS in the range of 9 to 12 were enrolled. The patients were randomly assigned to the treatment with simvastatin and placebo groups. The patients were evaluated according to GCS criteria at admission, discharge and 10 days after discharge as well as GOS criteria, one month, 3 months and 6 months after discharge.

**Results:**

No significant difference was observed between two groups, regarding the mechanism of injury, type and location of the lesion and complications. The comparison of the average temperatures showed that, the average temperature of patients treated with simvastatin was significantly lower. There was no significant difference between GCS and GOS of the two groups at all times. The comparison of GCS difference between the first and tenth days showed that the increase of GCS was higher in the group treated with simvastatin.

**Conclusions:**

The effects of statins on intracerebral hemorrhage have been confirmed, but few studies have been conducted on brain trauma. The present study revealed that though simvastatin caused no significant difference in GCS and GOS, but it had no harmful effects as well. It is recommended to conduct a study with a larger sample size and isolated groups.

**Keywords:**

Simvastatin, Patients, Traumatic brain injury


					Volume 4, Suppl. 1 Nov 2012
Publisher: Kermanshah University of Medical Sciences
URL: http://www.jivresearch.org
Copyright:
Congress of Iranian Neurosurgeons, 14-16 November 2012, Kermanshah, Iran
Paper No. 63
The effect of simvastatin on patients with traumatic brain
injury
Saleh Rasras a
, Farhad Rahbariyan a,*
, Mojtaba Khezri a
a
Department of Neurosurgery, Bojnord Hospital, Bojnord, Iran.
Abstract:
Background: Traumatic brain injury is one of the most common causes harmful to the health of society. Several studies
have been conducted on the treatment of traumatic brain injury. The protective effects of statins on neurons have
been demonstrated in numerous studies. The objective of the present study is to examine the effect of simvastatin on
the short-term and long-term results of consciousness in patients with brain trauma.
Methods: 66 patients with traumatic brain injury with GCS in the range of 9 to 12 were enrolled. The patients were
randomly assigned to the treatment with simvastatin and placebo groups. The patients were evaluated according to
GCS criteria at admission, discharge and 10 days after discharge as well as GOS criteria, one month, 3 months and
6 months after discharge.
Results: No significant difference was observed between two groups, regarding the mechanism of injury, type and
location of the lesion and complications. The comparison of the average temperatures showed that, the average
temperature of patients treated with simvastatin was significantly lower. There was no significant difference between
GCS and GOS of the two groups at all times. The comparison of GCS difference between the first and tenth days
showed that the increase of GCS was higher in the group treated with simvastatin.
Conclusions: The effects of statins on intracerebral hemorrhage have been confirmed, but few studies have been
conducted on brain trauma. The present study revealed that though simvastatin caused no significant difference in
GCS and GOS, but it had no harmful effects as well. It is recommended to conduct a study with a larger sample size
and isolated groups.
Keywords:
Simvastatin, Patients, Traumatic brain injury
* Corresponding Author at:
Farhad Rahbariyan: Department of Neurosurgery,Bojnord Hospital, Bojnord, Iran.Tel:09151672090, Email: dr.rahbarian@gmail.com, (Rahbariyan F.).

				

